# Children Reading to Dogs: A Systematic Review of the Literature

**DOI:** 10.1371/journal.pone.0149759

**Published:** 2016-02-22

**Authors:** Sophie Susannah Hall, Nancy R. Gee, Daniel Simon Mills

**Affiliations:** 1 School of Life Sciences, University of Lincoln, Lincoln, United Kingdom; 2 WALTHAM Centre for Pet Nutrition, Waltham on the Wolds, Leicestershire, United Kingdom; University of Tuebingen Medical School, GERMANY

## Abstract

**Background:**

Despite growing interest in the value of human-animal interactions (HAI) to human mental and physical health the quality of the evidence on which postulated benefits from animals to human psychological health are based is often unclear. To date there exist no systematic reviews on the effects of HAI in educational settings specifically focussing on the perceived benefits to children of reading to dogs. With rising popularity and implementation of these programmes in schools, it is essential that the evidence base exploring the pedagogic value of these initiatives is well documented.

**Methods:**

Using PRISMA guidelines we systematically investigated the literature reporting the pedagogic effects of reading to dogs. Because research in this area is in the early stages of scientific enquiry we adopted broad inclusion criteria, accepting all reports which discussed measurable effects related to the topic that were written in English. Multiple online databases were searched during January-March 2015; grey literature searches were also conducted. The search results which met the inclusion criteria were evaluated, and discussed, in relation to the Oxford Centre for Evidence Based Medicine levels of evidence; 27 papers were classified as Level 5, 13 as Level 4, 7 as Level 2c and 1 as Level 2b.

**Conclusion:**

The evidence suggests that reading to a dog may have a beneficial effect on a number of behavioural processes which contribute to a positive effect on the environment in which reading is practiced, leading to improved reading performance. However, the evidence base on which these inferences are made is of low quality. There is a clear need for the use of higher quality research methodologies and the inclusion of appropriate controls in order to draw causal inferences on whether or how reading to dogs may benefit children’s reading practices. The mechanisms for any effect remain a matter of conjecture.

## Introduction

Literacy skills have significant consequences to global health and economy. More than 796 million people in the world cannot read (approximately 15% of the population), resulting in world-wide costs of over USD $1 trillion a year, with the effects of illiteracy being very similar in developing and developed countries [1]. Poor literacy skills have substantial health and welfare implications for society, having been associated with reduction in: health outcomes, economic growth, social participation, self-esteem and hygiene, as well as increased accidents and job absenteeism [2]. It is clear that reading skills have wide-reaching implications. Likewise, in the educational environment the effects of literacy are not just relevant to performance in English lessons, but also have wider implications, determining successful academic learning in all subjects [3] and being associated with overall school enjoyment [4]. In the past decade there has been a worrying decline in children’s enjoyment, and therefore frequency, of reading [5]. Given that frequency of reading is directly related to reading attainment [3] it is essential that there are evidence-based interventions that increase children’s motivation, enjoyment and frequency of reading. Despite increasing Government awareness for the necessity of improving student’s motivation to read for pleasure [6] there is still no legitimised program to support this.

The first high profile programme to advocate children reading to dogs was established in 1999 by Intermountain Therapy Animals, who announced Reading Education Assistance Dogs (READ). Growing interest in reading to dog’s programmes such as READ is observed in frequent media reports and is reflected in the subsequent development of a number of initiatives around the world including (but not limited to), The Bark and Read Foundation (Kennel Club, UK), Caring Canines, Dogs Helping Kids, Read2Dogs, Classroom Canines (Delta Society, Australia), SitStayRead, Library Dogs, Tail Waggin’ Tutors (Therapy Dogs International), Reading with Rover, and All Ears Reading.

Proponents of READ postulate that reading to dogs helps motivate children to read by increasing relaxation and confidence, reducing blood pressure and offering a non-judgemental, safe environment in which to practice reading [7–8]. It is noted that READ (and similar organisations) do not supply evidence through control group comparisons to support these claims. However, in the wider literature there is evidence to suggest that improving reading motivation improves reading performance [9–11] indicating that if children are more motivated to read with a dog then this could improve their reading abilities. This may be especially important for students who struggle to read, because poor reading abilities are also associated with low reading motivation [4]. Also, below average readers often demonstrate increased reading anxiety; indeed, reading anxiety is a well observed form of ‘classical conditioning’ in the classroom environment [12]. For example, an initially neutral stimulus (e.g., reading out-loud) is repeatedly associated with a negative response (e.g., teacher judgement or peer ridicule), which results in the reader forming an association between reading and negative internal responses (e.g. anxiety, heightened emotions). Reading anxiety is common in children and is associated with physical symptoms, such as a reddening face, rapid breathing and tension headaches [13]. Evidence suggests that positive experiences can help the child to overcome negative associations and be more open to learning experiences [14] READ and similar programmes postulate that reading to a dog helps to overcome these (anxiety / motivation) roadblocks to developing reading expertise in the classroom. The silent companionship of a dog as a reading partner may allow the child to work at their own pace through reading challenges without fear of being judged. However, it is unclear what evidence exists to directly support the principles of READ (i.e., improved reading abilities through increased reading motivation and reduced reading anxiety).

The objective of this systematic review was to determine the scientific evidence base for the pedagogic effects of reading to dog’s programmes. We specifically focused on dependent measures such as reading speed and comprehension and/or learning behaviours in this evaluation of the available evidence. The aim was to objectively represent the current state of this developing field and in so doing we discuss all reports that investigated the reading abilities of children (under 16 years) in response to a reading to dog programme, even if they did not include a comparison group or use standardised tests. To illustrate the broad levels of scientific quality of the current data we report the finding in three stages; stage 1: results published in non-peer reviewed resources; stage 2: results published in peer-reviewed journals, but do not report original data; stage 3: reports of original data in a peer-reviewed journal, classified according to the hierarchy of evidence used by the Oxford Centre for Evidence Based Medicine (OCEBM) [15].

## Methods

The Preferred Reporting Items for Systematic Reviews and Meta-Analyses (PRISMA) guidelines were adhered to perform this systematic review [16] (S1 Appendix). No registered protocol exists for this review. The inclusion criteria for selection of articles included (a) literature that reports reading and/or behavioural effects of children (under 16 years, with or without a reading or developmental disability) reading to (in the presence of) dogs, and (b) articles written in English. With the aim of providing a comprehensive review of the current relevant literature we limited the number of specific restrictions for inclusion. We did not stipulate the nature (design details) of the intervention apart from ‘reading out-loud to dogs’, we included reports that collected data from a single time point, as well as studies that explore the effect of the intervention over time. We included reports that used both quantitative and qualitative measures of effect, including ad-hoc reports, making no stipulation on the use of outcome measures.

Literature searches were conducted in PubMed (1946-present), Science Direct (1946-present), American Doctoral Dissertations (1933–1955), Canadian Reference Centre (1901-present), Education Source (1900-present), ERIC (1966-present), Health Source: Nursing/Academic Edition (1952-present), MasterfilePremier (1921-present), PsycArticles (1984-present), PsychInfo (1987-present), Psychology & Behavioural Sciences Collection (1930-present), Social Sciences Full Text (1972-present), SocIndex with Full Text (1985-present) and Google Scholar (1946-present). In order to increase coverage, grey literature searches were conducted (search for relevant references used in the articles that were selected in stage 3). Table 1 contains the list of search terms used. Search terms were identified through analysis of commonly referred to key terms and titles in articles pertaining to reading to dogs. Full text articles were sourced for all references electronically, or via direct contact with the authors.

**Table 1:** Search Terms used in the Literature Search.

Dog(s) and reading	Reading assistance and dog(s)
Canine(s) and reading	Animal companionship and school
Dog(s) and child(ren) and reading	Animals(s) and school(s)
Dog(s) and reading and student(s)	Pet(s) and school(s)
Dog(s) and listening	Pet(s) and learning
Dog(s) and listening and child(ren)	Animal(s) and learning and education
Dog(s) and listening and student(s)	Dog(s) and learning and child(ren)
Dog(s) and school(s)	Dog(s) and cognition and child(ren)
Dog(s) and classroom(s)	Dogs(s) and child(ren) and performance
Dog(s) and literacy	

## Results

**Fig 1:**
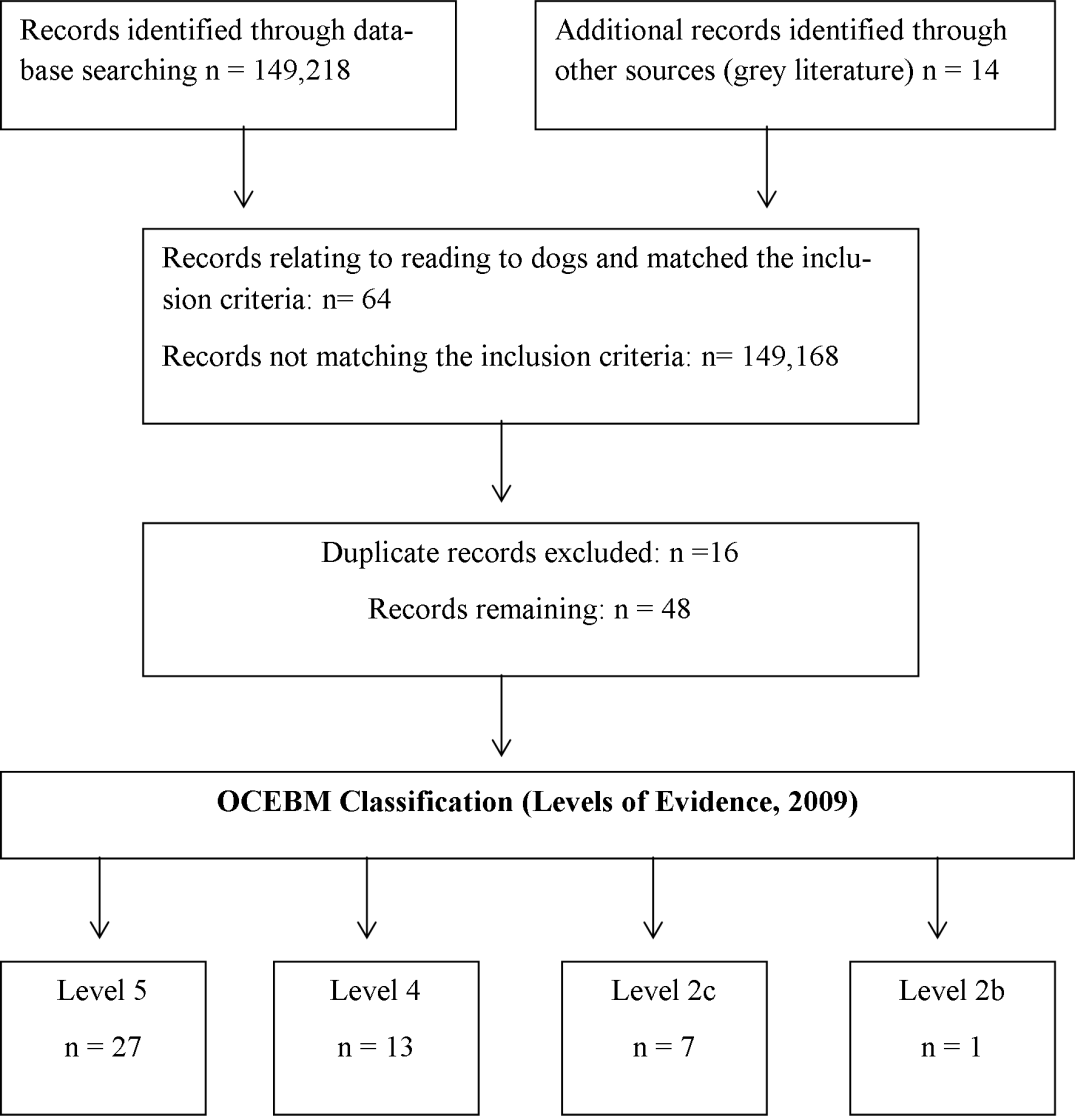
PRISMA (2009) Flow Diagram.

The initial literature searches, using the terms specified in Table 1 returned 149,218 results, with an additional 14 references obtained from grey literature searches contributing to a total of 149,232 references (Fig 1). After removing the topics that were not relevant to the aim of this paper and duplications of the maintained papers (S2 Appendix), 48 results were assessed against the OCEBM levels of evidence [15]. The OCEBM levels of evidence are designed to alert practitioners to the quality of evidence on which conclusions are based. The levels of evidence include: 1a: Systematic reviews (with homogeneity of variance) of Randomised Control Trials (RCTs). 1b: Individual RCTs with narrow confidence interval. 1c: All or none case series. 2a: Systematic reviews (with homogeneity) of cohort studies. 2b: Individual cohort studies, including low quality RCTs. 2c: Outcomes research, ecological studies. 3a: Systematic review (with homogeneity) of case-control studies. 3b: Individual case-control study. 4: Case series (and poor quality cohort and case control studies). 5: Expert opinion without explicit critical appraisal, or based on physiology, bench research or “first principles”. The authors (SSH, DSM) independently classified the papers with the OCEBM criteria and then met to discuss their ratings and resolve any discrepancies (step 1). NRG reviewed the ratings assigned (step 2), before submitting for independent review by two researchers not involved in any stage of the manuscript (step 3). No discrepancies occurred in stages 2 and 3. Discrepancies in stage 1 were not common, and involved a difference in rating by no more than 1 level. All discrepancies were resolved by reading the paper again to clarify understanding of the design and procedures.

Twenty-seven papers were ranked level 5, thirteen papers were ranked level 4, seven papers were ranked 2c, and one paper was ranked 2b. Specific details of the literature discussed can be found in Table 2 and Table 3.

**Table 2:** Evidence for the Value of Children Reading to Dogs.

RefNo.	First Author (year)	CEBM Rating	N:Type	Participant Age	Developmental Status	Effects on Reading Skills	Effects on Reading Behaviours
17	Bueche (2003)	5	NA:Opinion paper				Reduced reading anxiety, improved reading self-esteem
18	Burns (2014)	5	NA:Opinion paper				Reduced reading anxiety
19	Dunlap (2010)	5	NA:Opinion paper			Improved reading abilities	Greater reading confidence
20	Faver (2009)	5	NA:Opinion paper			Improved language and literacy skills	Greater reading confidence
21	Francis (2009)	5	NA:Opinion paper				Increased reading motivation & confidence
22	Hughes (2002)	5	NA:Opinion paper				Increased reading motivation & confidence
23	Inklebarger (2014)	5	NA:Opinion paper				Increased reading motivation, reduced reading anxiety
24	Jalongo (2005)	5	NA:Opinion paper			Improved reading abilities	Increased reading: motivation, self-esteem, enjoyment, feelings of support. Reduced reading stress
25	Kennel Club (2011)	5	NA:Opinion paper				Increased reading confidence
26	Klotz (2014)	5	NA:Opinion paper			Improved reading abilities	Increased reading: confidence, motivation, engagement. Reduced reading anxiety
27	Lane (2013)	5	NA:Opinion paper			Improved reading abilities	Increased reading motivation & confidence
28	Pillow-Price (2014)	5	NA:Opinion paper			Improved reading abilities	Increased reading: confidence, self-esteem. Reduced reading anxiety
29	Shannon (2007)	5	51:Survey	Guardians of 8 yr-olds			Increased reading motivation and confidence
30	Shaw (2013)	5	NA:Opinion paper			Improved reading abilities	Increased intrinsic reading motivation, increased reading engagement
31	Siegel (2004)	5	NA:Opinion paper			Improved reading abilities	Increased reading confidence, reduced reading anxiety
32	Snider (2007)	5	NA:Opinion paper			Improved reading ability	Improved reading confidence
33	Truett (2014)	5	NA:Opinion paper				Increased reading confidence, reduced reading anxiety
34	U Tenn Vet (2015)	5	NA:Opinion paper				Increased reading motivation
35	Friesen (2009)	5	NA:Opinion paper				Importance of multi-sensory experiences. Improved emotional, social & behavioural effects
36	Friesen (2010)	5	NA:Opinion paper				Increased feelings of social and emotional support
37	Friesen (2012)	5	NA:Opinion paper			Improved reading abilities	
38	Jalongo (2004)	5	NA:Opinion paper			Improved reading abilities	
39	Black	5	NA:Opinion paper			Reading rate improved by 24 words/minute	
40	Konarski (no date)	5	1:Case study	6 years	Autistic	Improved oral reading fluency (by 3 sounds) and non-sense word fluency (by 4 sounds)	Improved recreational and academic reading attitude
41	Gallatin (2014)	5	4:Interview & 13:Survey	Teachers of children 7–11years	Reading disability and typically developing readers		Improved confidence and motivation to read
42	Fisher (2014)	5	1	9 years	Disengaged reader	Improved reading accuracy and reading comprehension on Neale Analysis of Reading Ability	
43	Grigore (2014)	4	3	7–8 years	Autistic		Increased social initiations from the children
44	Heyer (2007)	4	6:Intervention 0:Control	Grades 2–4(7–9years)	Below average	Slightly increased reading abilities	Increased confidence and love for reading
45	Intermountain Therapy (2009)	4	12:Intervention 0:Control	Not recorded	Struggling readers	11/12 participants improved up to 4 reading grades	
46	Kaymen (2005)	4	4:Intervention 0:Control	Grade 3 teachers (8 years)	Not recorded		Improved attitude
47	Lloyd (2014)	4	11:Intervention 0:Control	5–11 years	At risk readers	Improved reading grades	Improved attitudes, increased confidence, co-operation & attendance
48	Newlin (2003)	4	15:Intervention 0:Control	Grade 2 (7 years)	Below average readers	Improved reading ability by minimum of two grades	
49	Walsh	4	5: Intervention 0:Control	6–7 years	Reading disabilities	Improved reading fluency (visual inspection)	Increased reading confidence and engagement
50	Griess	4	4: Intervention 0:Control	Grades 3–5 (8–10 years)	Learning disabilities	Spent more time reading	Improved reading motivation & enjoyment for reading
51	Smith (2010)	4	11:Intervention 0:Control	6–12 years	Home schooled	Reading fluency improved by 30%	
52	Martin	4	10:Intervention 0:Control	5–9 years	Not recorded	Improved scores on Test of Reading Comprehension (TORC) and Measures of Academic Progress (MAP)	
53	Bassette (2013)	4	3	7–11 years	Emotional/ behavioural disabilities		Increased on-task behaviours during reading: Behavioural Observation of Students in Schools (BOSS)
54	Moore (2013)	4	71:Repeated Measures	Grade 3 (8 years)	Not recorded		Changed implicit theories of human reading ability
55	Paradise (2007)	4	98:Intervention 19:Control	Grades 1–5 (6–10 years) Teachers	Not recorded	Improved identifying, exploring, defining, analysing, predicting, summarising	Improved reading: attitude, enthusiasm & self-esteem
56	Booten (2011)	2c	17:Intervention 15:Control	Grade 5 (10 years)	Not recorded	No significant differences	No significant differences
57	Lenihan (no date)	2c	9:Intervention 9:Control	Grade 2 (7 years)	Not recorded	Reading abilities declined more in control group	Reading attitude decreased more in control group
58	Peterson (2008)	2c	29:Intervention 26:Control	Grade 7 (12–13 years)	Not recorded	No significant differences	
59	Smith (2009)	2c	152:Intervention 98:Control	Grade 2 (7 years)	Low income families	Improved reading fluency	
60	Treat (2013)	2c	9:Intervention 8:Control	Grades 2–5 (7–10years)	Learning disabilities	Improved reading fluency, accuracy and comprehension	Improved reading self-perception and reduced reading anxiety
61	Wohlfarth (2014)	2c	12:Repeated-measures	6–7 years	German speaking	Improved: Correct words, punctuation & comprehension–recordings of reading	
62	Friedmann (1983)	2c	38:Repeated measures	9–5 years	Not recorded		Blood pressures were lower when reading to a dog
63	LeRoux (2014)	2b	27:Dog 24:Adult 26:Teddy 25:Control	7–13 years	Poor readers	Improved reading accuracy and reading comprehension in dog group	

**Table 3:** Evidence for the Value of Children Reading to Dogs (continued).

RefNo.	First Author (year)	Reading Measure	Behavioural Measure	Intervention (if applicable)	Length of Intervention	Duration of Sessions
17	Bueche (2003)			R.E.A.D	Not recorded	Not recorded
18	Burns (2014)			R.E.A.D	Not recorded	Not recorded
19	Dunlap (2010)				Not recorded	Not recorded
20	Faver (2009)				Not recorded	Not recorded
21	Francis (2009)			Library Therapy Dog		One per week
22	Hughes (2002)				Not recorded	Not recorded
23	Inklebarger (2014)				Not recorded	Not recorded
24	Jalongo (2005)			R.E.A.D	Not recorded	Not recorded
25	Kennel Club (2011)			Bark&Read		
26	Klotz (2014)			R.E.A.D	Not recorded	Not recorded
27	Lane (2013)			Canine Assisted Reading Programmes	Not recorded	Not recorded
28	Pillow-Price (2014)			SitStay&Read	Not recorded	Not recorded
29	Shannon (2007)			Library Dog	Not recorded	Not recorded
30	Shaw (2013)			R.E.A.D	Not recorded	20–30 minutes
31	Siegel (2004)				Not recorded	Not recorded
32	Snider (2007)				Not recorded	Not recorded
33	Truett (2014)			Paws for Reading	Not recorded	Not recorded
34	U Tenn Vet (2015)			Ruff Reading	Not recorded	Once per week
35	Friesen (2009)				Not recorded	Not recorded
36	Friesen (2010)				Not recorded	Not recorded
37	Friesen (2012)			R.E.A.D	Not recorded	Not recorded
38	Jalongo (2004)			R.E.A.D	Not recorded	Not recorded
39	Black			R.E.A.D	Not recorded	Not recorded
40	Konarski (no date)	Oral Reading Fluency (ORF), Nonsense Word Reading Fluency (NWF) & Dynamic Indicators of Basic Early Literacy Skills (DIBELS)	Elementary Reading Attitudes Scale (ERAS)		5 weeks	Not recorded
41	Gallatin (2014)	Teacher report	Measure of Academic Progress Assessment	R.E.A.D	Teacher report	Measure of Academic Progress Assessment
42	Fisher (2014)	Neale Analysis of Reading Ability		Classroom Canines	8 weeks	1 hour (4 students: 15 mins reading)
43	Grigore (2014)				15 minutes	6 sessions per condition
44	Heyer (2007)			R.E.A.D	16 weeks	20 minutes
45	Intermountain Therapy (2009)			R.E.A.D	8 weeks	20 minutes
46	Kaymen (2005)			SHARE a book	Not recorded	Not recorded
47	Lloyd (2014)			Classroom Canine	18 weeks	Not recorded
48	Newlin (2003)			Carolina Canines for Service Project	1 academic year	20 minutes
49	Walsh (2014)				10 minutes (3 times/week)	6 weeks
50	Griess (2010)	Informal Reading Inventory			13 weeks (2 weeks holidays)	20 minutes
51	Smith (2010)	Dynamic Indicators of Basic Early Literacy Skills (DIBELS		Sit,Stay&Read	8 weeks	1 hour (shared)
52	Martin (2001)	Test of Reading Comprehension (TORC) and Measures of Academic Progress (MAP)		R.E.A.D	15 months	20 minutes
53	Bassette (2013)		Behavioural Observation of Students in Schools (BOSS)		4 weeks	30 minutes
54	Moore (2013)				16 weeks	45 minutes (group)
55	Paradise (2007)			Canine Assisted Reading		30 minutes
56	Booten (2011)	Pearson-Scott Foreman	Respect & Protect (School behavioural management plan)		Not recorded	3 days/week in class
57	Lenihan (no date)	Curriculum Based Measurement (CBM)	Elementary Reading Attitudes Scale (ERAS),	R.E.A.D	5 weeks	30 minutes
58	Peterson (2008)	Degrees of Reading Power (DRP)			5 days	20 minutes
59	Smith (2009)	Dynamic Indicators of Basic Early Literacy Skills (DIBELS)		Sit,Stay&Read	8 weeks	1 hour (shared)
60	Treat (2013)	Grey Oral Reading Test (GORT) & Basic Reading Inventory (BRI)	No standardised (own scales)		10 sessions	10–15 minutes
61	Wohlfarth (2014)	Audiotaped. Scoring protocol designed			Not recorded	Not recorded
62	Friedmann (1983)		Blood Pressure		Not recorded	Not recorded
63	LeRoux (2014)	Neale Analysis of Reading Ability			10 weeks	20 minutes

### OCEBM Classification Level 5

Level 5 Evidence is “Expert opinion without explicit critical appraisal, or based on physiology, bench research or first principles” [15]. The majority of literature that met the search criteria was classified as Level 5 evidence (n = 27, 56%). Typically, these documents detailed ad-hoc reports of the effects of reading to a dog as evidenced by classroom teachers, or dog handlers. Many of the documents associated reading to a dog with behavioural changes, particularly, greater motivation to read to a dog, improved confidence when reading to a dog, and reduced signs of anxiety when reading to a dog [17–34]. Some documents also indicated that children reported feeling more supported when reading to a dog [22, 25, 35–38]. References to improvements in actual reading abilities were also noted, but not as frequently as changes to behavioural processes [19, 20, 24, 26–28, 31, 32, 37, 39–42]. Only a small number of documents made reference to any standardised tests that were used when making these judgements [40, 42]. A disengaged reader was reported as showing improvements on the Neale Analysis of Reading Ability (NARA) after taking part in a reading to a dog programme [42], and a child with autism spectrum disorder was reported as improving on the Dynamic Indicators of Early Literacy Skills (DIBELS) and Elementary Reading Attitudes Scale (ERAS) after completing a reading to a dog intervention [40]. However, these case studies did not use any control measures, or included a case series, and therefore were categorised as Level 5 evidence. These studies show some promise that reading to a dog can benefit children’s reading abilities by altering key behavioural process which may be important in contributing to an optimal learning environment, specifically, by increasing reading motivation and confidence, and reducing reading anxiety.

### OCEBM Classification Level 4

Level 4 evidence is classified as “Case series, and poor quality cohort and case control studies”.

Thirteen papers (27%) of the documents were classified as Level 4 evidence. Six of the 13 studies were case series studies that followed the progress of a small group of children as they took part in a reading to a dog intervention, but did not use standardised measures to assess the effects of the programme [43–48]. Four of the studies were based on children with reading disabilities, or below average reading skills [44, 45, 47, 48], one study involved children with autism spectrum disorder [43], and one study did not report the developmental status of their population [46]. These case studies reported improved reading abilities when reading to a dog, as measured by teacher opinion or reading grade [44, 45, 47, 48]. Alterations to reading behaviours were also documented, including, improved reading confidence [44, 47], greater attention when reading, improved attendance to reading lessons [47], more positive attitude to reading [46] and increased social initiations when reading [43].

Five of the 13 papers classified as Level 4 evidence used standard tests to measure the effects of reading to a dog on a case series group of children, but did not include control conditions. The tests used included, the AIMSweb Curriculum Based Measurement [49], the Informal Reading Inventory [50], the Grey Oral Reading Test [51], the Test of Reading Comprehension and Measures of Academic Progress [52] and the Behavioural Observation of Students in School [53]. Three of the studies measured the effects of reading to a dog on a-typically developing children, with reading disabilities [49], learning disabilities [50], or emotional and behavioural problems [53]. The studies reported that reading to a dog has a positive effect on reading abilities, improving reading fluency [49, 51] and enhancing reading comprehension [52]. Evidence of positive alterations to behavioural processes were also documented, including, improved reading motivation [50], reading confidence [49], reading attitude and enjoyment [49–51] and increased engagement and on-task behaviours [49, 53].

Two of the 13 papers classified as Level 4 evidence implemented control procedures, in terms of using a repeated measures design [54], and a control group condition [55], but did not use standard measures to assess the effects of reading to a dog. The first of these papers reported that after reading to a dog children changed their implicit theories of reading ability, which may lead to a positive attitude to reading lessons [54]. The second paper reported that children who read to a dog improved on specific reading skills, including the ability to identify, explore, define, analyse, predict and summarise when reading, in comparison to a group of children who did not read to a dog [55].

The studies classified as Level 4 evidence support the conclusions made based on the Level 5 evidence; reading to a dog may enhance a child’s reading environment, by increasing reading motivation and confidence, reducing anxiety, increasing task engagement and reading attitude. There is some evidence to suggest that after children take part in a reading to a dog programme they improve their scores on reading tests; however, such conclusions need to be assessed in relation to appropriate control conditions for this to be taken with a degree of confidence.

### OCEBM Classification Level 2c

Level 2c evidence is “Outcomes research”. The search documents have been classified as Level 2c evidence if they clearly state the outcome of reading to a dog, using standard tests and in relation to a control group or condition, using consistently applied reference standards (without which the data would be classified as Level 3b). No evidence was classified as Level 3a, because no systematic reviews were identified.

Seven papers (15%) were classified as Level 2c evidence for the effects of children reading to dogs. All of the papers used a standard or objective approach to measure the effects of reading to a dog. None of the papers reported effect sizes and some failed to report either descriptive or inferential statistics. Five of these seven papers assessed the effects of reading to a dog in comparison to a control group [56–60]. One of the five papers assessed the effects of reading to a dog with children with learning disabilities [60]; the remaining four did not specify the developmental status of their population. Two of the five papers that used a control group comparison found no significant effects of reading to a dog on performance on the Degrees of Reading Power test [58] and a Pearson-Scott Foreman reading test [56]. However, the first mentioned study only assessed reading ability after taking part in a five-day intervention [58], and the other failed to record the duration of the intervention [56].

Two of the five papers reported a statistically significant increase in reading abilities as measured by the Dynamic Indicators of Basic Early Literacy Skills [59] and Grey Oral Reading Test [60] in children who took part in a reading to a dog programme. Only one of these studies included the reading performance of the control group in the statistical analysis, and used a co-variate analysis to control for reading performance at baseline [59]. This study reported that the end of program totals for oral reading fluency, after being adjusted for baseline performance were 78.38 (mean reading fluency) for the intervention group and 71.04 for the control group. The authors do not state whether these are high or low scores on this test.

The second study referred to conducted a t-test analysis of pre and post intervention reading ability separately for the intervention and control group [60]. This study reported an increase in mean reading performance on the GORT for the intervention group (reading rate increase of: 1.78 (Pre-test: 5.44 ± 2.18; Post-test: 7.22 ± 2.33; *p =* 0.007), reading accuracy increase of: 3.34 (Pre-test: 5.66 ± 1.80; Post-test: 9.0 ± 1.58; *p* = 0.001), reading fluency increase of: 2.88 (Pre-test: 5.0 ± 1.87; Post-test: 7.88 ± 2.08, *p* = 0.002]), reading comprehension increase of: 1.89 (Pre-test: 7.44 ± 1.59; Post-test: 9.33 ± 1.32, *p* = 0.000), oral reading quotient increase of: 14.16 (Pre-test: 77.5 ± 10.46; Post-test: 91.66 ± 9.22, *p* = 0.001); NB: Descriptive statistics on ranges were not presented in the paper)). However, mean figures were not provided for the control group, therefore, even a visual inspection of differences between the control and intervention group cannot be made. Furthermore, without the use of case matching it is important that appropriate statistical techniques are employed to control for differences in reading levels at baseline between intervention and control groups.

One study assessed the effects of reading to a dog over school summer vacation. This small pilot study found no statically significant differences as a result of the programme, however this could be due to the small sample size (9 children in the intervention group and 9 children in the control group). Indeed, the authors observed that children in the control group (who read to a human alone) showed trends to reduced reading ability and poorer reading attitudes over the summer vacation, than children who were reading to a dog. No effect sizes or mean values were reported to support this statement, although visual inspection of the graphed data indicates that the control group showed a reduction in reading ability (words per minute) that was double that shown by the intervention group. The authors also noted that no children dropped out of the intervention group, but attendance was reduced over time in the control group [57].

The final two out of the seven papers classified at Level 2c used a repeated measures design to assess the effects of reading to a dog. One paper reported the effects of 12 children reading to a dog, in comparison to reading to a human alone, using repeated measures, cross over design [61]. They report a clear documentation of how they assessed reading ability, despite not using a standardised reading test. They observed that when children read to a dog they performed better on tasks including word recognition (Dog: 96.45±0.79; Mean±SD, Control: 94.83±1.67) recognitions of punctuation marks (Dog: 82.84±7.60; Control: 70.73±8.76), and use of line breaks (Dog: 79.66±4.50; Control: 71.93±7.97). These improvements were not statistically significant.

The second paper which used repeated measures, cross over design measured physiological responses (blood pressure) of 38 children when they were with a dog and engaging in reading and when they were resting, and when they were not with a dog and reading and resting [62]. The presence of the dog reduced blood pressure when the children were reading and when they were resting. Although the study did not assess what effect this change in anxiety had on reading performance, it does represent the first study to apply objective, physiological, measures to assess the effects of reading to a dog on child anxiety levels.

These studies, classified at Level 2c, support the conclusions drawn from Level 5 and Level 4 evidence. Reading to dogs may bring about changes to children’s behavioural processes, which has a positive impact on the environment in which reading is practiced and in turn facilitates reading performance.

### OCEBM Classification Level 2b

Level 2b evidence includes “Individual cohort studies, including low quality randomised control trials.”

One paper (2%) returned from the search procedures was classified as Level 2b evidence. Le Roux et al. [63] randomly assigned 102 third grade students from one school, who were identified as poor readers, to one of four conditions. The study does not document whether any statistical procedures were used to calculate the required sample size, and instead appears to be based on a convenience based sampling of the population. Twenty-seven students read to a dog (through Pets as Therapy teams), 24 read to an adult, 26 read to a teddy bear with an adult and 25 students were in a control group that did not include a reading intervention. Reading ability was measured using the Neale Analysis of Reading Ability at baseline (Time 1), after the 10-week programme (Time 2) and eight weeks later (Time 3). The intervention ran for 10 weeks and each session lasted for 20 minutes. The authors state that there was no significant difference between reading abilities of the four groups at baseline, but no inferential or descriptive statistics are reported to support this statement, nor were appropriate statistical techniques employed to control for differences between the populations. Indeed, visual inspection of their figures (p665) shows that reading comprehension was higher in the dog group than the other groups at baseline [63]. No significant effects of group were reported between the time points. However, over all time points reading rate was significantly better in the dog group (7.94 ± 0.96) compared to the teddy bear group (7.45 ± 0.79) with a medium effect size reported (η² = 0.09). Both reading accuracy and reading comprehension were better in the dog group (reading accuracy: 7.73 ± 1.13; reading comprehension: 7.29 ± 0.13) compared to the adult group, teddy bear group (reading accuracy: 7.21 ± 0.78; reading comprehension: 6.59 ± 0.80) and control group (reading accuracy: 7.28 ± 0.77; reading comprehension: 6.74 ± 0.83) with large effect sizes (reading accuracy: η² = 0.13; reading comprehension: η² = 0.15) Demographic information for each student was collected, but not reported in the paper. It is important that studies report demographic information to evaluate to whom reading to dogs may be most beneficial for. This study shows promise for reading to dog programmes for struggling readers, but does not consider the potential mechanisms involved in this process.

## Discussion

In this discussion we first consider to what extent the existing evidence base, reported in this review paper, supports the pedagogical value of reading to dogs. We also discuss potential mechanisms that may be involved when reading to a dog. We draw upon the literature presented here as well as including broader literature from the animal-assisted intervention (AAI) field, to suggest how reading to a dog may affect reading performance.

The 48 studies that met the search criteria described positive effects of children reading to dogs. In particular, the papers evidenced improvements to the children’s behavioural processes, which may improve the environment in which reading is practiced, and therefore lead to better reading performance. However, the quality of the evidence on which these conclusions are drawn is low, with the majority categorised at the lowest level (Level 5) on the OCEBM criteria [15]. Much of the evidence is based on ad-hoc reports that have not been through a peer-review process. Conclusions are typically based on inferences from small sample sizes. Additionally, studies do not use blind scoring, fail to consider longitudinal durability of the changes observed, do not sufficiently control for baseline scores or appropriately randomly allocate children to intervention or control groups. Only one report claims to have conducted a randomised control trial [63], but given the relatively small sample size in each group, the quality of evidence remains low.

It is clear that future research in this area should prioritise using standardised measures to assess the effects of reading to a dog. Few studies use measures that have clear documentation of their validity and reliability, indeed few studies even use standard measures as used in educational practice, and instead rely on subjective judgements, such as teacher opinion. Furthermore, no studies use standardised scales to assess the relationship between reading performance and behavioural processes, which is important to help understand how reading to a dog programme may benefit children. Such an evaluation would also help us to identify which children may be most likely to benefit from taking part in these programmes and therefore lead to the development of clinically and economically effective practices. In a similar light, in order to develop codes of conduct of best practice and a reading to dog’s curriculum it is essential that future reports and scientific investigations document details of the intervention, including the number of sessions, duration of the sessions, details of the dog(s) and demographic variables of the children (e.g. age, disability status, and experience with dogs).

Despite the criticisms that can be levelled at the existing evidence base, there is clear documentation that reading to a dog has the potential to bring significant improvements to children’s reading abilities, and therefore deserves further investigation. Based on figures provided by informal communications with registered school dog organisations, a trained dog and handler can assist a child to read for one year for around £60 (GBP). If volunteer handlers are involved, as is often the case, this figure reduces to around £16 (GBP). When considering the current global costs of illiteracy [1, 2], reading to dog’s programs may represent a unique, cost effect strategy which could be implemented into a broader reading curriculum. However, before the practice of reading to dogs is adopted into mainstream education it is vital that the practice is subject to greater scientific scrutiny in order to evaluate how these programmes may benefit children, and if these gains are of clinical significance. Such evaluations are also important in developing specific reading curricula. Much of the current practice in reading to dogs occurs without specific guidelines, or evidence of documented lesson plans. For educators and policy makers to recognise the value of reading to dog’s programmes it is important that curricula are developed which have clear and measurable learning goals.

### How might Reading to Dogs Improve Reading Abilities?

**Fig 2:**
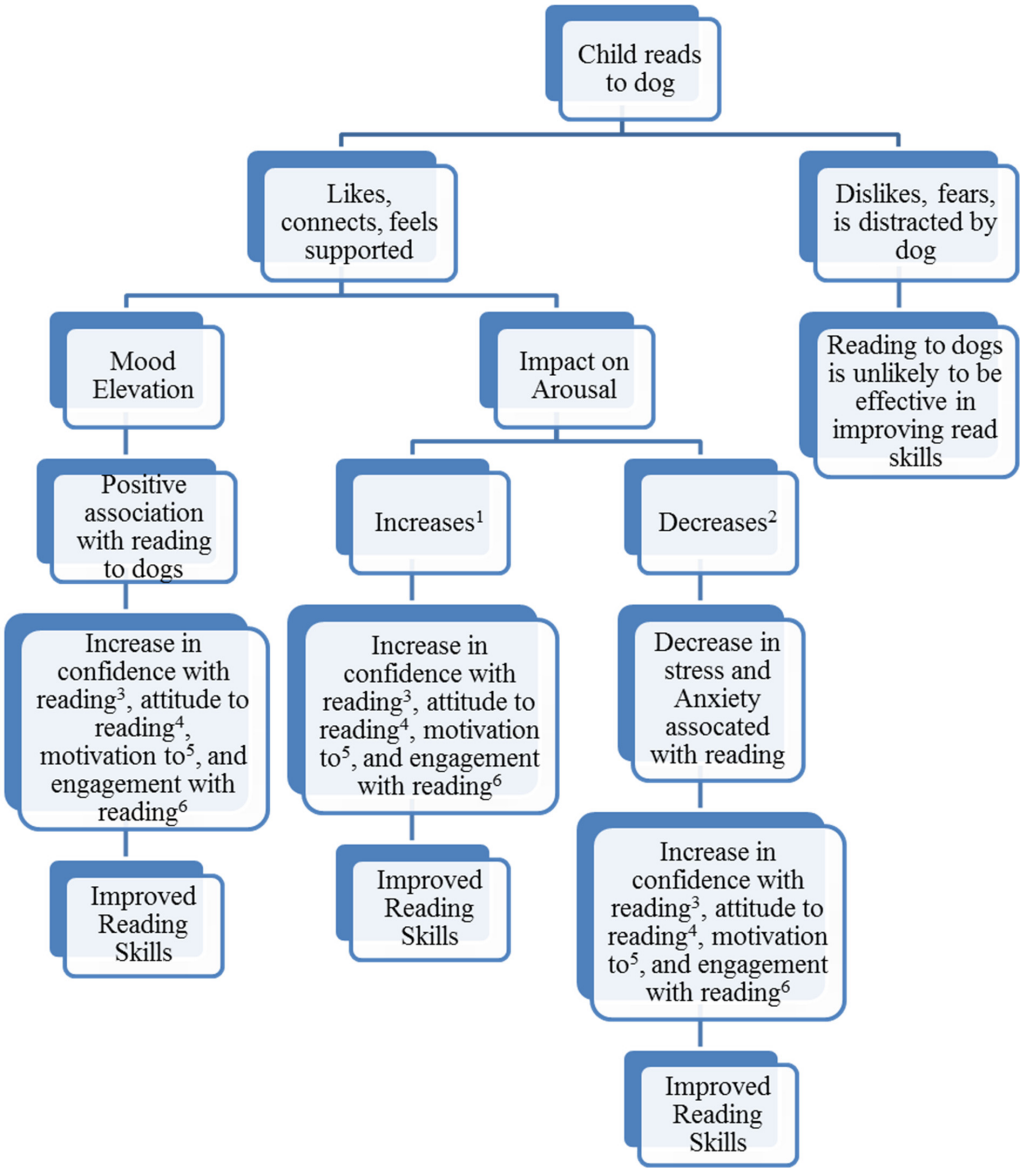
An Illustration of how Reading to a Dog may Influence Reading Performance. ^1^Increased arousal can heighten cognitive performance on some tasks [64, 65]. Whether increased our decreased arousal results in optimal performance is typically determined by the individual and the nature of the task (see Individual Zone of Optimal Functioning) [66, 67] ^2^Reading anxiety (over-arousal) can impair children’s reading performance in the classroom [12] by negatively impacting on cognitive processes involved in reading, including problem-solving and self-regulation [68, 69]. ^3^ A reader’s self-concept or confidence in their reading ability determines reading practices [70], by influencing the amount of time and the degree of effort which is put into reading [71]. There is a positive relationship between reading self-concept and reading achievement [72, 73]. The dynamics of the relationship between self-concept and achievement is still debated it is thought to be reciprocal, with greater achievement raising student’s self-concept, as well as higher self-concept leading to improved academic achievements [74]. ^4^There is an association between reading attitudes and reading performance in children, with those who have a positive attitude doing better in reading tests than those who have a negative attitude [75, 76]. Attitudes towards reading are thought to influence attainment by determining reading behaviours (e.g. frequency of reading) [70]. ^5^In reading studies motivation is often discussed in terms of intrinsic motivation (motivated from within; e.g., curiosity to read, enjoyment of the experience) and extrinsic (motivated by external factors; e.g., to get a good grade) [77, 78]. Although both factors are thought to play in role in influencing reading behaviours, intrinsic motivation is thought to be the key determiner [78–80]. ^6^Engagement in reading is often associated with motivation. According to Guthrie and Wigfield [81] reading motivation is an interaction with text that is both motivated and strategic, engaged reading is related to reading comprehension success, and engaged reading can be improved by instructional practices that use motivational and cognitive strategies.

Many of the papers listed in this review make reference to students demonstrating a change in behavioural processes during reading to dog’s programmes (Table 2). Such a change is taken as an indication that reading to dogs may improve the wider learning environment for children, which may improve reading performance. In Fig 2 we provide a graphical description as to how reading to a dog may influence behavioural processes, enhancing the learning environment and ultimately leading to improved reading performance.

The processes which are outlined in Fig 2 are evidenced in the wider field of human-animal interactions as being processes which can change in the presence of an animal.

#### Top-down processes

There is evidence to suggest that animal-assisted therapy (AAT) with children with Autism Spectrum Disorder (ASD) produces increased displays of positive attitudes and feelings, as evidenced by increased smiling and laughing [82, 83] and decreased problematic behaviours [83]. Similar improvements have been observed in classroom situation [84], which has led some to suggest that a classroom dog may improve a child’s attitude to school [85]. However, these classroom studies, like the reading to dog’s studies are based on small-scale subjective observations and do not include a control group. Gee and colleagues have conducted a series of controlled laboratory-based investigations to explore the impact of the presence of a dog on a range of children’s cognitive and motor tasks [86–90]. These studies found that with a dog, compared to without a dog, young children perform motor tasks faster without sacrificing accuracy [86], require less assistance with a memory task, potentially demonstrating improved concentration [87], require fewer instructional prompts to complete imitation tasks [88], show fewer errors on a cognitive task [89], and show improved object recognition [90]. These studies imply that a dog may improve the motivation of children to engage and accurately complete set tasks.

#### Confidence

McConnell et al. [91] provide one of the most recent studies to suggest that animals can improve confidence (defined here as including the constructs of self-esteem and self-concept); with adult pet owners scoring higher on Rosenberg’s [92] scale of self-esteem than non-pet owners. However, it is difficult to determine a causal effect, in that people self-select for pet ownership status so it is possible that people with high self-esteem may be more likely to choose to own a pet, rather than pet ownership increasing low self-esteem. There appears to be little research that has explored how pet dogs may affect child confidence, yet this could prove to be an important mechanism in determining how dogs may benefit children’s educational achievement. Nonetheless, one study of 130 adolescents showed that those who owned a pet have greater self-esteem than those who did not [93], but again, it is difficult to infer causation. Only a single conference paper shows a possible causal effect of the dog over time (as opposed to population selection bias) with the addition of a classroom pet improving self-esteem in children over a nine-month period in comparison to a control group [94].

#### Anxiety

The moderating effects of dogs on human anxiety and stress are a re-occurring theme in AAT literature [95–97]. Although ad-hoc reports indicate that anxiety reducing effects are observed in the context of classroom dogs to date there is insufficient evidence of appropriate quality to support this claim, but neither is there evidence to deny it. Evidence from physiological indicators of anxiety have the advantage of being free from experimenter bias, but often relate to general arousal rather than a specific state and show a lack of consensus as to whether dogs have an excitatory effect, a calming effect, or no effect. For instance, Somervill et al. [98] reported a significant increase in the blood pressure and heart rate of 17 children diagnosed with attention-deficit hyperactivity disorder (ADHD) after handling a dog. It is noted that the authors did not include a familiarisation period with the dog, indeed the child only spent one five-minute session with the dog, and the child was not given a structured task to do to focus their attention. Other studies suggest that the presence of a dog may reduce physiological parameters of stress, decreasing blood pressure when reading to a dog [62] and reducing cortisol awakening in children with insecure attachment [99] and with autism [100]. These effects were observed in comparison to a control condition. A recent study showed that physical contact with a dog during a stressful working memory task did not significantly alter the heart-rate variability of undergraduate students in comparison to contact with a stuffed toy or another human [101], suggesting that dogs do not reliably influence humans physiological stress responses. It is noted that that this study used adult rather than child participants which leaves open the possibility that dogs may be more effective at reducing child, compared to adult, physiological reactions. A further possibility is that dogs only reduce physiological arousal in a specific population. Comparisons of children’s skin conductivity responses showed that the presence of a dog reduced arousal in children with ASD, but increased arousal in typically developing children in one study [102]. It is clear that further research is needed to establish whether children’s reading to dogs modulates physiological measures of arousal in general or anxiety specifically, and whether decreases (e.g. a calming effect) or increases (e.g. heightened arousal and attention) are most conducive to the learning environment.

#### Social Support

The social support hypothesis is often mentioned in the study of animal companionship, with evidence suggesting that animals offer social support themselves, as well as acting as ‘social lubricants’ to facilitate interactions with other humans [81, 91, 103–105]. Indeed, ad-hoc reports suggest that the non-threatening, non-judgemental presence of a dog improves children’s feelings of support during reading [24]. Although the influence of dogs on young children’s perception of social support is largely under-explored, Beetz et al. [99] reported that children, aged 7–11 years, experience greater levels of support in a stressful social situation in the presence of a dog than a friendly human. Further research is needed to investigate these effects in educational environments.

#### Engagement

The notion of animals affecting our attention and engagement is related to the commonly cited biophilia hypothesis in animal companionship research. This states that humans have developed to attend to living systems, including animals, due to an innate drive to be close to other living things, perhaps for safety and/or because of the positive feelings associated with learning about the living world [106, 107]. Evidence to support this is observed in studies which show that adults show faster identification of visual changes to scenes that contain animals or humans than those that contain inanimate objects, when controlling for dynamicity of the scenes [108]. More specifically, a study has shown that children preferred to interact with, and showed greater attention to, animals in comparison to stimulating toys [109]. Additional evidence supporting the effect of dogs to improve a child’s attention and engagement comes from a clinical study highlighting a reduction in problematic attention/deficit-hyperactivity symptoms in children diagnosed with attention/deficit-hyperactivity disorder (AD/HD) when participating in a canine-assisted intervention [110]. The studies reported by Gee and colleagues provide some evidence to suggest that dogs may help typically developing children to engage in structured tasks, similar to that required in classroom lessons [86–89]. However, children’s willingness to respond to animals, or desire to be close to them, is likely to be mediated by individual differences and life experiences as well as an innate drive. Those individuals with less positive perceptions or experiences with animals are less likely to show improvements through animal interactions [111, 112]. Given the frequent negative portrayals of animals in the media (e.g. regarding dog bites) the effect of negative experience may be a more important issue that warrants careful consideration in both practice and experimental design.

In summary, the evidence suggests that reading to a dog has a positive impact on the learning environment in which reading is practiced. However, based on our classifications using the OCEBM the quality of this evidence is poor. We recognise that despite using an objective classification system we cannot rule out bias in our judgement when assigning classifications. With the aim of reducing this potential bias all members of the team met to discuss classifications. Additionally, we sought independent opinions from two additional research professionals; no discrepancies occurred. We are unable to provide effect sizes for the majority of the results discussed in this paper, due to poor design and reporting procedures. For this reason, and because of the lack of Randomised Control Trials (RCTs) identified through the searches, it was not possible or relevant to conduct a meta-analysis on the data. As well as representing a critique point for this systematic review it highlights the current state of the art, with need for greater scientific rigour in this field. Nonetheless, there is some evidence from wider HAI field to support the conclusion. Drawing upon the wider field provides some convergent validity for the practice of reading to dogs, but the conclusions should still be interpreted with caution. Although outside of the scope of this paper, much of the existing HAI research can also be subject to a number of criticisms. These problems further highlight the need for future studies in this field to include rigorous controls and high quality scientific designs such as large scale randomised control trials in order to systematically explore, and understand, the impact of HAI.

## Summary and Conclusion

This paper presents the first systematic review exploring the value of HAI in educational practice, specifically the practice of reading to dogs. The review highlights the plausibility of reading to dogs to bring improvements to children’s reading abilities and beneficially alter behavioural and emotional processes which may be important aspects in creating a learning environment to best cultivate reading skills.

However, there is a clear need for more rigorous investigation in this area. Large scale randomised control trials, with longitudinal examinations of effects are needed to improve the quality of the evidence base of the research and to further our theoretical understanding of the underlying mechanisms impacted by the dog presence as well as the effective implementation of such programmes. It is important that studies use appropriate sample sizes to enable confidence in the detection of meaningful effects. Additionally, there is a need for practitioners and researchers to work together to evaluate specific reading to dog’s curricula in order to ensure any benefits are maximised and studies comparable.

## Supporting Information

S1 Appendix PRISMA Checklist (DOCX)

S2 Appendix Duplications Removed from the Review (DOCX)
