# Supplementary material for: Children Reading to Dogs: A Systematic Review of the Literature
Source: PLoS One. 2016 Feb 22;11(2):e0149759. doi: 10.1371/journal.pone.0149759 (PMC4763282; doi:10.1371/journal.pone.0149759)
Supplement: S2 Appendix — (DOCX) [file pone.0149759.s002.docx]

**S2 Appendix. Duplications Removed from the Review**

The following 16 papers appeared more than once in the search procedures, but were only reviewed once. These papers are the 16 duplicates that are referred to in Figure 1.

1. Black S. Sit, stay, and read. Am Sch Board J. 2009;196(12): 36–37
2. Booten AE. Effects of animal-assisted therapy on behavior and reading in the classroom. Undergraduate thesis, Marshall University. 2011.Available: <http://mds.marshall.edu/cgi/viewcontent.cgi?article=1023&context=etd>. Accessed 25 March 2015
3. Friesen L. Potential for the role of school-based animal-assisted literacy mentoring programs. Lang Lit. 2010;12(1): 21-37
4. Gallatin E. How Reading Education Assistance Dogs (READ dogs) in an elementary intervention program are perceived by teachers and students. School of Education Paper 24. 2014. Available: <http://digitalcommons.hamline.edu/hse_all/24/>. Accessed 16 March 2015
5. Griess JO. A canine audience: the effect of animal-assisted therapy on reading progress among students identified with learning disabilities. Doctoral thesis, University of South Florida. 2010. ISBN-978-1-1242-6712-8
6. Hughes K. See spot read. Public Libraries. 2002;41(6): 328–330
7. Jalongo MR. ‘‘What are all these dogs doing at school?’’ Using therapy dogs to promote children’s reading practice. Child Educ. 2005;81(3): 152–158
8. Jalongo MR, Astorino T, Bomboy N. Canine visitors: The influence of therapy dogs on young children's learning and well-being in classrooms and hospitals. Early Child Educ J. 2004;32(1): 9-16
9. Kaymen MS. Exploring animal-assisted therapy as a reading intervention strategy. Master’s thesis, Dominican University of California, CA. Available: <http://files.eric.ed.gov/fulltext/ED490729.pdf>. Accessed 16 March 2015.
10. Konarski J. More than just a class pet: Reading therapy dogs and young readers with autism. Report. Available: <http://jkonarski.cmswiki.wikispaces.net/file/view/More+Than+Just+A+Class+Pet-+Reading+Therapy+Dogs+And+Young+Readers+With+Autism.rtf>. Accessed 16 March 2015
11. Le Roux MC, Swartz L, Swartz E. The effect of an animal-assisted reading program on the reading rate, accuracy and comprehension of grade 3 students: A randomized control study. Child Youth Care Forum. 2014;43(6): 655-673
12. Pillow-Price K, Yonts N, Stinson L. Sit, stay, read: Improving literacy skills using dogs! Dimensions of Early Childhood. 2014;42: 5-9. Available: [http://www.southernearlychildhood.org/upload/pdf/SitStayRead_D42_1.pdf. Accessed 25 March 2015](http://www.southernearlychildhood.org/upload/pdf/SitStayRead_D42_1.pdf.%20Accessed%2025%20March%202015).
13. Walsh AE. Impact of animal assisted therapy on oral reading fluency of second graders*.* Doctoral thesis, State University of New York at Fredonia. 2014. Available: <https://dspace.sunyconnect.suny.edu/bitstream/handle/1951/64521/Alison_Eckert_Masters_Project_May2014.pdf?sequence=1&isAllowed=y>. Accessed 16 March 2015
14. Wohlfarth R, Mutschler B, Beetz A, Schleider K. An investigation into the efficacy of therapy dogs on reading performance in 6-7 year old children. Hum Anim Int Bull. 2014;2: 60-73
15. [Smith CE. An analysis and evaluation of sit stay read: is the program effective in improving student engagement and reading outcomes? Doctoral thesis, National-Louis University, Chicago, IL. 2009. Available: http://digitalcommons.nl.edu/ cgi/viewcontent.cgi?article=1032&context=diss](file://C:\Users\Hall\Documents\D.Sophies%20Research%20Plans\2.WORD_Animal%20Assisted%20Literacy\LITERATURE\Writing%20Review\Smith%20%20CE.%20An%20analysis%20and%20evaluation%20of%20sit%20stay%20read:%20is%20the%20program%20effective%20in%20improving%20student%20engagement%20and%20reading%20outcomes?%20Doctoral%20thesis,%20National-Louis%20University,%20Chicago,%20IL.%202009.%20Available:%20%20http:\\digitalcommons.nl.edu\%20cgi\viewcontent.cgi?article=1032&context=diss). Accessed 16 March 2015
16. Smith MH, Meehan C. All ears reading program and home-schooled youth. Final report: UCD Veterinary Medicine Extension, University of California, CA. 2010. Available: <https://www.yumpu.com/en/document/view/11807739/all-ears-readingtm-tony-la-russas-animal-rescue-foundation>. Accessed 16 March 2015.
